# Evaluation of Children’s Anxiety Level in Relation to a Dental Visit/Treatment and Their Parents’ Dental Fear

**DOI:** 10.3390/jcm12206691

**Published:** 2023-10-23

**Authors:** Pia-Merete Jervøe-Storm, Lisa Patricia Peters, Katrin Bekes, Miriam Fricke, Søren Jepsen

**Affiliations:** 1 Department of Periodontology, Operative and Preventive Dentistry, University of Bonn, Welschnonnenstrasse 17, 53111 Bonn, Germany; soeren.jepsen@ukbonn.de; 2 Independent Researcher, Lortzingstraße 38, 53332 Bornheim, Germany; lisapatricia.peters93@gmail.com; 3 Department of Paediatric Dentistry, University Clinic of Dentistry, Medical University of Vienna, Sensengasse 2a, 1090 Vienna, Austria; katrin.bekes@meduniwien.ac.at; 4 Independent Researcher, Glogauer Straße 28, 53117 Bonn, Germany; mi.fricke@gmx.de

**Keywords:** children, anxiety, CFSS-DS, pediatric dentistry, parents, caries

## Abstract

The patients’ fear of the dentist plays an important role in the everyday life of a dentist. The anxiety level of children in relation to dental treatment/visits and to their parents’ dental fear was evaluated in three different centers. Assessments of a modified CFSS-DS (mCFSS-DS) were performed by questionnaire with 60 children and their parents. Children’s dmft/DMFT scores, age and gender were evaluated in relation to the parents’ perception of their child’s anxiety levels. For statistical evaluation, Kruskal–Wallis and Wilcoxon tests as well as Spearman’s correlation coefficient (Spearman) were used. The significance level was set at 0.05. There were no significant differences regarding children’s mCFSS-DS between the three centers (*p* = 0.398, Kruskal–Wallis). The parents’ mCFSS-DS scores correlated significantly with their children’s mCFSS-DS scores (*p* = 0.004, Spearman). However, the mean mCFSS-DS score of the children was significantly higher than the mean score of parents’ perception of their child’s anxiety (*p* = 0.000, Wilcoxon). The age of the child had an influence on the mCFSS-DS score (*p* = 0.02, Kruskal–Wallis) but neither the children’s gender (*p* = 0.170, Kruskal–Wallis), nor the dmft/DMFT showed an impact (*p* < 0.725, Spearman). Although a positive correlation was found between the results of the children’s and parents’ questionnaire, many parents underestimated the anxiety level of their children.

## 1. Introduction

The patients’ fear of the dentist plays an important role in the everyday life of a dentist. Dealing with anxious patients is time-consuming and stressful for the dentist [1,2]. Especially with children, it is a problem known worldwide. Dental anxiety in children may prevent them from making prophylactic regular visits to the dentist, resulting in a higher risk of caries and a loss of good oral health [3]. Another aspect is the risk of the progression of dental fear into adulthood [4]. Dental fear in adults has a negative correlation with their oral-related quality of life (OH-QoL) [5]. So, to prevent later problems with poor oral health, it is important to understand children’s fear or anxiety [2].

The prevalence of dental fear or anxiety (DFA) in childhood is estimated to be between 5.7% and 46.8% in different populations [3]. Mild anxiety is to be expected as an experience, but if the anxiety is disproportionate to the actual threat, it becomes a problem and may interfere with a possible need for dental treatment [6]. According to a review from 2007, dental fear is described as a normal emotional reaction to various stimuli in the dental setting; dental anxiety is referred to as a concern that something terrible is going to happen in connection with dental treatment; and dental phobia represents a severe subset of dental anxiety [6]. For the examination of dental anxiety in children up to 14 years of age, the CFSS-DS (Children’s Fear Survey Subscale-Dental Subscale) is the most commonly used measure [6,7].

Some studies found differences in DFA levels between different age groups [8,9] whereas other studies showed a significant difference in DFA between genders [3,8]. In contrast, studies using the CFSS-DS or a visual-analogue scale for dental fear (DF-VAS) were not able to demonstrate such a difference for genders [10,11,12] or for age [11,13,14].

A relationship between poor oral health and DFA was shown [3,15,16]. Likewise, DFA in context with the degree of caries was demonstrated [11,16,17]. For the assessment of caries prevalence, dmft/DMFT is used. dmft/DMFT adds the number of decayed, missing, or filled teeth together (deciduous dentition: dmft; permanent dentition: DMFT) [18]. Dmft/DMFT levels were higher in children aged 6 to 12 years with DFA than in children with low anxiety scores [11].

Often, children’s fear or anxiety develops in early childhood and is established before the first visit to the dentist [19]. A recently published study showed that the later a child becomes familiar with visits to the dentist or with a low frequency of visits, the higher the risk of developing DFA [9]. Another investigation found a strong correlation between parental dental fear and children’s fear [16]. In a Swedish study including 210 parent/child pairs, the extent to which parents could adequately assess their child’s DFA was found to be poor, especially in a group with high levels of DFA [20].

The causes of fear may be linked to parents’ influence on children’s behavior at the dentist and their attitude towards it [2,6,15]. Various factors must be considered in order to assess DFA in children. The developmental stage of the childr plays an important role [21]. If a questionnaire is used to assess the level of DFA, it should be ensured that the child is able to understand and answer it adequately. The child should have sufficient comprehension and language skills and a related level of cognitive development to ensure the validity of the answers [21].

The aim of the present study was to investigate the following research questions:

- Is there a difference between the anxiety scores of the children who visit a pediatric dentist’s office, a university dental clinic, or a general dental office that does not focus on pediatric dentistry?
- Is there a correlation between the parent’s expected anxiety scores for their child and the child’s own anxiety score?
- Is there a difference in anxiety between the genders of the children?
- Is there a correlation between the children’s anxiety score and their age?
- Is there a correlation between the anxiety score and the DMFT/dmft?

## 2. Materials and Methods

### 2.1. Study Design

The present study was conducted as a cross-sectional study. Children aged 6–14 years, each with one legal guardian (further on denominated as “parent”), participated in the study as a couple. Exclusion criteria for the children were:

- conditions associated with frequent visits to a medical practitioner
- pain or swelling of oral origin
- mental retardation
- cleft lip and palate
- generalized mineralization disorders
- orthodontic treatment

Written information was provided for both the parent and the child with all relevant facts about the study, while anonymity was also guaranteed. For the child, this information was written in child-friendly language and, if necessary, also read aloud. Dialogue or questioning of the child was only possible after obtaining signed consent from a parent. The child’s consent was given either verbally or in writing, depending on the age of the child. The parents’ consent was given in writing. Participation was completely voluntary and independent of further treatment measures in the respective treatment centers. Both the child and the parents had the option to withdraw their consent at any time. The rejection of a child overrode the consent of the parents.

The study participants came from a general dental practice with no special focus on pediatric dentistry (GP), a pediatric dental practice (SPP) and the pediatric section of the Department of Periodontology, Operative and Preventive Dentistry of the University Hospital Bonn (UHB), all three centers in Bonn (Germany) and the surrounding area. The two practices and the university participated with 20 pairs, each of a child and its parent. The study was approved by the university’s ethics committee, University of Bonn, Germany (ID: 257/17; date of approval 23 November 2017). The study was conducted in accordance with the European directives and ICH Harmonized Tripartite Guideline E6: Note for Guidance on Good Clinical Practice, CPMP/ICH/135/95 Step 5 (http://www.ema.europa.eu/ema/, accessed 12 March 2021), as well as in compliance with the Declaration of Helsinki (2013 Brazil).

### 2.2. Questionnaire

The focus of the study was the questionnaire, which was intended to reflect the children’s fears when visiting the dentist. Both the children and their parents filled out almost the same questionnaire. The basis for this is the “Dental Subscale of Children’s Fear Survey Schedule (CFSS-DS)” [7]. In 1982, Cuthbert and Melamed [7] modified the earlier Fear Survey for Children [22] from 1968 and developed the Children’s Fear Survey Schedule (CFSS), which served as the basis for this study. The reliability of the scale has several times been proved to be satisfactory [6,23,24,25].

Cuthbert’s original questionnaire contains fifteen questions related to various dental terms and instruments. Each item can be rated on a scale of 1 (not afraid at all) to 5 (very afraid), so the total score ranges from 15 to 75. For the present study, the fifteen questions from Cuthbert and Melamed’s version [7] were translated from English into German, with only fourteen questions being adopted. The question of fear of injections was eliminated on the advice of a specialized pediatric dentist. The first thing a child is afraid of when going to a dentist is an injection [26]. In order not to upset the child, this question was not asked. Thus, the total score was between 14 and 70.

The questionnaire was designed in such a way that one was filled out by the child and a second by the parents. It is important to mention that the task of the parents was to fill out the questionnaire from the supposed point of view of their child, and not from their own feelings.

To be able to assess the attitude of parents toward their own dental examination or treatment, they were additionally asked one question about their own feelings on a scale from 1 (no fear at all) to 5, where score 2 was connected with a slight fear, increasing to score 5 (a lot of fear). This should help later to allow further comparisons between parents and children.

The disadvantages of a questionnaire for recording dental treatment anxiety include the subjectivity of the individual respondents and the indirect influence of the interviewer. In this respect, an external person conducted the survey. This is recommended, especially in the case of children who might be strongly influenced by their parents [16]. The questionnaires were always completed in the presence of the same investigator; both the parent and the child completed a questionnaire separately. This means that neither the child nor the parent were present when the other party filled out the questionnaire. The investigator was not allowed to interfere in the process of answering the questions; only in cases of uncertainty about the meaning of the question was an objective explanation allowed. The forms were provided with an anonymized identification number and the examination date of the subject.

### 2.3. Dental Examination

There were two indices to assess the children’s caries experience. One was the dmft used for primary teeth: decayed, missed and filled teeth (dmft) and the other was the DMFT for permanent teeth, based on the World Health Organization (WHO) [18]. In the present study, the dmft and the DMFT were both collected because of the transitional dentition in most of the children. The investigator did not clinically examine the children; this was performed by the same experienced person in each center. According to the WHO categorization of caries values, dmft/DMFT values of zero were considered no caries, less than 2.7 was considered low, 2.7 to 4.4 was considered moderate, and 4.5 and more were considered high caries experience [18].

### 2.4. Data Collection

The results of the study were treated anonymously. At the beginning, general information such as age, date of birth, gender and ethnicity of both parents and children was asked. Thereafter, the modified questionnaire (mCFSS-DS), based on the CFSS-DS [7], was completed by both parties. Furthermore, the children’s DMFT/dmft was calculated and noted. The data were analyzed anonymously on the basis of the allocation of a study number, which could not be associated with the birthday or other personal data. The questionnaires of the parents and the children could be linked to each other anonymously on the basis of the coding and stored separately.

### 2.5. Statistical Evaluation

The following null hypotheses were tested:

- There is no difference between the anxiety scores of the children who visit a pediatric dentist’s office, a university dental clinic, or a general dental office that does not focus on pediatric dentistry.
- There is no correlation between parents’ expected anxiety scores for their child and the child’s own anxiety score.
- The genders of the children have no influence on the anxiety score.
- There is no correlation between children’s anxiety levels and their age.
- There is no correlation between the anxiety score and their DMFT/dmft.

The software IBM SPSS 27.0 software (IBM, Armonk, NY, USA) was used for the analysis. The significance level for the tests was set at 0.05. The individual anxiety score was determined for each subject. Previous studies classified scores below 32 (42.7% of a maximum sum of 75 on CFSS-DS) as clinically irrelevant [27]. Krikken and co-workers [2] also used a low or high anxiety threshold of 32. In the present study, this was modified according to the 14 questions, giving a mCFSS-DS < 30 (42.8%) as low anxiety and a mCFSS-DS ≥ 30 as high score. The threshold of 30 (42.8% of a maximum sum of 70 of mCFSS-DS) used in the present study corresponds to the threshold of the other studies [2,27].

The Kruskal–Wallis test was used to compare the mean values of anxiety scores in terms of gender, age, dmft/DMFT and visits to different practices and clinics. Furthermore, the chi-square or Fisher-exact test was used to investigate a possible dependency between children and parents. The Spearman correlation coefficient was calculated to illustrate each correlation.

## 3. Results

### 3.1. Study Population

In total, sixty children aged 6–13 years and sixty parents participated in the study from February to December 2018. Thus, a total of 60 pairs, including a total of 120 people, were included in the present study. From each center, 20 pairs were included (Table 1). Mostly, the mother was accompanying the child (48; 80%). No child was visiting the respective center for the first time.

### 3.2. Questionnaires

To check the reliability of the mCFSS-DS, the internal consistency was determined with the help of Cronbach’s alpha. The scale of the mCFSS-DS had good internal consistency (0.857, Cronbach’s alpha).

Table 2, and, respectively, Table 3 reflects the answers from the children and the parents. It is interesting to note that all participants in all three centers have the highest score in the present mCFSS-DS for question 12: “having to go to hospital”. Here, a mean score of 3.2 to 3.5 for children and a mean score of 2.5 to 3.1 for parents was calculated.

### 3.3. Comparisons

#### 3.3.1. Comparison ‘Questionnaires Children’ vs. ‘Questionnaires Parents’

There were no significant differences regarding the anxiety scores between the groups of children who visited a specialized pediatric dentist’s office (SPP), a university hospital pediatric section (UHB), or a general dental practice (GP) (*p* = 0.398, Kruskal–Wallis).

In the present study, 21 children reached the level of a high anxiety score (≥30). There were only eight children above the second threshold, as originally suggested by ten Berge and coauthors [27]. Therefore, in the present study, it was decided to use only a mCFSS-DS sum of 30 as a threshold for low or high anxiety and not a second one of 36. The median mCFSS-DS of the children was higher than parents’ assessment of their child’s supposed anxiety (children: mean 26.8; SD 7.64; parents: mean 22.5; SD 6.86). Out of 60 pairs, 44 parents (73.3%) correctly assessed their child’s anxiety as either high (≥30) or low (<30) (Table 4). Of the remaining 16 pairs, three parents (5%) rated their child’s anxiety as high (range 31–35), but the child himself or herself had a low assessment (range 17–26). In contrast, thirteen parents (21.7%) rated the child’s anxiety as low (range 15–26), but the child rated himself or herself with high anxiety (range 30–52). Of these thirteen children, eight were boys. The mean discrepancy in the group ‘HA_c_ vs. LA_p_’ (Table 4) between the child’s mCFSS-DS and the parent’s mCFSS-DS was 14.1 (SD 6.53; range 6–32). Nevertheless, there was a significant correlation between children’s mCFSS-DS and parents’ mCFSS-DS (Spearman’s correlation coefficient 0.371, *p* = 0.004). The level of parents’ own fear had no influence on children’s mCFSS-DS (*p* = 0.117, Wilcoxon), but on parents’ perception of their child’s level of anxiety (*p* = 0.003, Wilcoxon).

Altogether, a positive predictive value (PPV) of 26.5% (95% CI 15.4 to 41.3) and a negative predictive value (NPV) of 27.3% (95% CI 7.3 to 60.7) was observed. However, these values do not assess the quality of the mCFSS-DS, but rather how parents correctly assessed their children’s anxiety (Table 5).

The results of the present study confirmed that children and their parents have different perceptions of fear. In this study, children had a significantly higher mean anxiety score (*p* = 0.000, Wilcoxon). However, in the university hospital, this could not be confirmed (GP: *p* = 0.011; SPP: *p* = 0.020; UHB: *p* = 0.089, Wilcoxon). The influence of the ethnic background of the children on the mCFSS-DS was not possible to calculate because of the heterogeneous distribution (Table 1).

#### 3.3.2. Comparisons ‘Gender’ vs. ‘mCFSS-DS’

The gender of the children did not have any influence on the mCFSS-DS score in the present study (*p* = 0.170, Kruskal–Wallis). The mCFSS-DS scores are reflected in Table 6, where the values for each gender subdivided by age are listed.

#### 3.3.3. Comparisons Age vs. mCFSS-DS

The age distribution of children in three centers did not differ significantly (*p* = 0.258, Kruskal–Wallis). However, the actual age of the child had a significant influence on the mCFSS-DS (*p* = 0.02, Kruskal–Wallis). The age intervals of the children as shown in Table 1 had no significant correlations with the mCFSS-DS (*p* = 0.157, Kruskal–Wallis). The parents’ perception of their child’s anxiety was also not dependent on the child’s age (*p* = 0.465, Kruskal–Wallis). The age of the child at its first visit did not show any correlation with the level of mCFSS-DS of the child (Spearman’s correlation coefficient −0.010, *p* = 0.937).

#### 3.3.4. Comparisons dmft/DMFT–Index vs. mCFSS-DS

Twenty-seven children (45%) aged 6–13 years were caries-free. Eight (36.4%) of the 6–7 year olds, 10 (62.5%) of the 8–9 year olds and 4 (26.7%) of the 10–11 year olds had no caries. Five of the six 12-year-old children included (83.3%) were caries-free.

Due to their age, all of the included children presented with a transitional dentition. The distribution of dmft/DMFT is shown in dependence on the investigation center (Table 7) and the age of the included children (Table 8). Hereby, it is noticeable that the patients of the university hospital had the highest dmft/DMFT score, followed by the specialized pediatric practice. Not surprisingly, the 6–7 year-old children had the highest dmft score, while the group of 10–11-year-old children had the highest DMFT (Table 8).

There was a significant difference between the three centers for the dmft. The children examined in the university hospital had a higher number of decayed, missing and filled teeth in the primary dentition than the other two centers (*p* = 0.001, Kruskal–Wallis). This was not found for the permanent dentition (*p* = 0.158, Kruskal–Wallis).

There was no correlation between dmft/DMFT and the mCFSS-DS in the present study (dmft: Spearman’s correlation coefficient −0.046, *p* = 0.725; DMFT: Spearman’s correlation coefficient 0.008, *p* = 0.950). A high mCFSS-DS (≥30 in the present study) was found in 21 (35%) children. Of these 21 children, 9 (42.9%) had no caries at the same time (dmft/DMFT = 0). Corresponding to this, 17 children (43.6%) of 39 children (65%) with a low mCFSS-DS had no caries (dmft/DMFT = 0).

The influence of the ethnic background of the children on the level of caries was not possible to calculate because of the heterogeneous distribution (Table 1).

## 4. Discussion

### 4.1. Main Findings

In the present study, a modified CFSS-DS (mCFSS-DS) questionnaire was filled out by 60 children. Another similar questionnaire was filled out by one of their parents. It was designed to assess the parent’s perception of their own child’s level of anxiety. The present study was performed in three different settings: a general dental practice with no special focus on pediatric dentistry (GP), a pediatric dental practice (SPP) and the pediatric section of the Department of Periodontology, Operative and Preventive Dentistry of the University Hospital Bonn, Germany (UHB).

There were no significant differences regarding the anxiety scores between the groups of children who visited the three centers (*p* = 0.398). The mean anxiety score of the children was significantly higher than the mean score of parents’ perceptions of their child’s anxiety (*p* = 0.000). But the mCFSS-DS scores of the parents correlated significantly with the mCFSS-DS scores of the children (Spearman’s correlation coefficient 0.371, *p* = 0.004). Of the fourteen questions in the mCFSS-DS, the highest score was found for question number 12: “Having to go to the hospital” by all 120 participants. The gender of the children did not have any influence on the mCFSS-DS score (*p* = 0.170). The actual age of the child had an influence on the mCFSS-DS score (*p* = 0.02) but not on the answers from the parents (*p* = 0.465). There was no correlation between the level of caries and the mCFSS-DS in the present study, neither for dmft (Spearman’s correlation coefficient −0.046, *p* = 0.725) nor for DMFT (Spearman’s correlation coefficient 0.008, *p* = 0.950).

### 4.2. Material and Methods

The number of pairs of children and their parents included was relatively lower than in other studies. However, in these studies, school settings were used to recruit children [3,9,14,15]. In the present study, contact with the children who came to their appointment was made spontaneously. This is a limiting factor for the study, but it reflects the “real world”. The presence of a mother or father to answer the second ‘parent’s questionnaire’ was hereby warranted, which was an essential item of the present study. Moreover, the child should be monitored for the level of caries and not be in orthodontic therapy. The latter restricted the inclusion in the study considerably, together with the high number of rejections from the children and the parents both. All this, together with time pressure, limited the possibility of collecting child–parent pairs willing to participate. The scarce time for the questionnaires is probably due to the organization of dental care in Germany, where children are mainly cared for in private practices. In a private practice, the additional time or willingness of children or parents to answer questionnaires, etc., may be limited due to the child’s concerns about the upcoming examination or from the parent’s side. The children in the present study were referred to the pediatric specialist or the pediatric dental section of the university hospital because of difficult treatment options. Another limitation is the inclusion of a specific population of children in Bonn, Germany and its surroundings. However, to our knowledge, this is the first study to compare three different centers in assessing children’s dental anxiety related to parents’ perceptions of their own child’s anxiety and their own feelings about dental treatment.

### 4.3. Comparisons with Other Studies

In the present study, the original 15-question CFSS-DS questionnaire was modified to 14 questions on the recommendation of a specialized pediatric dentist. The question of injections should not be discussed with the child. Nevertheless, the reliability of the mCFSS-DS was calculated to be good, with an internal consistency of 0.857 (Cronbach’s alpha). The reliability was in line with the results of other studies [2,23,25].

A study on 2114 children used not only a threshold for anxiety at 32 but also at 39 [27]. Below 32 the CFSS-DS score was seen as a non-clinical range. Between 23 and 38, a range was defined as a borderline range. A clinical range was defined as 39 and higher. The first cut-off was also adapted in the present study, but due to the low number of participants, the second cut-off was not used in this study. Krikken and coworkers [2] used ‘32’ as a cut-off for a low or high level of anxiety. In the present study, this was modified with cut-off scores of <30 (low anxiety) and ≥30 (high anxiety) for the mCFSS-DS.

Forty-four parents were able to correctly identify their child’s feelings in connection with a visit to the dentist. But 16 parents (26.7%) had a poor understanding of their child at the visit to the dentist. In 13 cases, the child was frightened, but the parent did not perceive this.

In a study from the Netherlands in a classroom setting with 326 children and a home response from 167 of their mothers, this was not found [2]. In that study, parents tended to estimate their children’s dental fear higher than their children. But in the present study, the child and parent were in the dental practice, and with all its noises and smells, which may also trigger anxiety, the child’s score was higher than the parent’s score. In a university setting, 100 children’s anxiety during dental local anesthesia was investigated. No difference between parents’ and children’s fear was found [28]. In that study, all questionnaires were filled out only by the parents and not by the child itself.

A strength of the present study was the fact that the child was not influenced by its parents while filling out the questionnaire. Filling out the CFSS-DS alone provided higher reliability and validity compared with other studies [3]. In some studies, the questionnaire was completed with the parents’ assistance or by their parents solely at home or by telephone, leading to inaccurate outcomes [3,25,27].

Some international studies have shown gender effects when using the CFSS-DS. Frequently, a higher level of anxiety could be detected, especially in girls [3,13,19,29,30]. This result can be attributed mainly to the different development of girls and boys. It is not uncommon for girls to show their emotions. They are often portrayed as the weaker ones in society, which is why it is not uncommon for many that girls are more likely to show their fears than boys [27]. In the present study, no significant gender effect could be shown, neither in general nor specifically related to age groups. These results are also found in other studies [10,25]. In the present study, the non-existent gender effect may be attributed to the small group of subjects or to the social circumstances from which the children come.

Other studies included between 134 and 2144 children and yielded a mean CFSS-DS score between 21.2 and 27 [2,3,8,13,20,23,27]. Despite the comparatively small number of participants in the present study (60 children and 60 parents), the mean CFSS-DS score of 26.8 agreed well with the results of the other studies.

The results of the present study suggest that dental fear or anxiety decreases with increasing age. This was also found by Cuthbert and Melamed in their original publication of the CFSS-DS [7]. They also showed that children aged 6–7 years have the highest levels of CFSS-DS. The present study did not support these findings by sorting the children in age intervals. However, using the actual age of the included children instead of age intervals, the results of Cuthbert and Melamed within the mentioned limitations could be confirmed. There was a high mCFSS-DS score for 13-year-olds, but there was only one child in this group. Therefore, this value is not meaningful in this context. Other studies found that anxiety decreases with age or that there is no significant association between anxiety development and age [13,16,29,31,32].

In the latest published version of the fifth German Oral Health Survey (Deutsche Mundgesundheit Studie V) from 2016 [33], 1468 12-year-old children were monitored for caries; 81.3% were free of caries in the permanent dentition (DMFT 0). In the present study, twenty-seven children (45%) aged 6–13 years were caries-free. But the children in the present study visited the practice mainly because of a treatment need. Fortunately, five of the six 12-year-old children included (83.3%) were caries-free in the permanent dentition, which is very consistent with the nationwide findings. A longitudinal study over a two-year follow-up investigated the influence of caries experience on CFSS-DS. They found that children with poor oral health exhibited a higher risk of dental fear and reverse [17]. In the present study, there was no significant correlation between dmft/DMFT and anxiety scores.

Since the number of participants in the present study was small compared to other studies, it would be interesting to repeat the study with a larger number of participants. Applying a second questionnaire to both children and parents after treatment might be an interesting option for such a follow-up study. Understanding and managing a child’s anxiety can help optimize long-term oral health. Some adults suffer from dental fear as a reminder of previous treatment experiences with significantly impaired oral health-related quality of life.

## 5. Conclusions

Within the limitations of the present study, it was shown that not all parents were able to assess the level of anxiety of their child correctly. Even though a positive correlation between children’s and parents’ questionnaires was found, sixteen of sixty parents rated their child’s anxiety incorrectly. Of these, thirteen parents rated their child’s anxiety too low. This reflects how important it is to ask the child about its feelings and not only rely on parents’ perceptions of their child. Using such a questionnaire for the child, the dentist can better adapt and individualize the child’s treatment based on the child’s information. The dentist should not rely exclusively on the statements of the accompanying parents but rather use a combination of both for the child’s benefit.

## Figures and Tables

**Table 1:** Demographic data of the included participants (children and parents); *n*.

		GP	SPP	UHB	Total
Children					
	Gender				
	Boy	9	11	11	31
	Girl	11	9	9	29
	Age				
	6–7 years	7	6	9	22
	8–9 years	6	5	5	16
	10–11 years	4	5	6	15
	12–14 years	3	4	0	7
	Mean (years; SD)	8.7 (1.98)	9.2 (2.08)	8.1 (1.70)	8.6 (1.95)
	Ethnical background				
	Caucasian	18	17	7	42
	Oriental	0	3	9	12
	Asian	0	0	4	4
	African	2	0	0	2
Parents					
	Gender				
	Male	1	3	8	12
	Female	19	17	12	48
	Age				
	20–30 years	3	2	3	8
	31–40 years	7	7	7	21
	41–50 years	6	10	7	23
	51–60 years	4	1	3	8
	Mean (years; SD)				40.6 (7.95)

GP: general dental practice; SPP: specialized pediatric practice; UHB: university hospital Bonn. There were no significant differences between the three groups in regard to children’s age or gender (*p* > 0.05).

**Table 2:** Result of the mCFSS-DS questionnaire for the child (highest score in bold and underlined).

How Do You Feel at the Thought of …	GP		SPP		UHB	
	Mean	SD	Mean	SD	Mean	SD
1. Dentists	1.7	0.80	2.1	0.97	2.0	1.28
2. Doctors	1.7	0.73	1.7	0.75	1.7	0.75
3. Having somebody examine your mouth	2.3	1.07	2.1	0.79	2.0	1.03
4. Having to open the mouth	1.5	0.69	1.5	0.51	1.8	1.07
5. Being touched by dentist or nurse	1.6	0.94	1.9	0.88	2.1	1.36
6. Having somebody look at you	1.6	0.68	2.0	0.86	1.8	1.29
7. The dentist checking your mouth	1.8	1.12	1.7	0.81	1.6	1.10
8. The sight of the dentist checking somebody’s mouth	1.6	0.68	1.8	0.95	1.7	1.26
9. Noise during treatment	1.9	0.89	2.3	1.26	2.4	1.04
10. Having somebody put instruments in your mouth	1.8	0.93	2.3	1.22	2.3	1.56
11. Getting water in your mouth during treatment	1.3	0.57	1.9	0.99	2.0	1.21
12. Having to go to the hospital	**3.5**	1.24	**3.5**	1.36	**3.2**	1.58
13. People in white uniform	1.5	0.83	1.7	0.73	1.4	0.75
14. Having dentist or nurse cleaning your teeth	1.3	0.73	1.6	0.75	1.9	1.14

GP: general practice; SPP: specialized pediatric practice; UHB: university hospital Bonn. There were no significant differences between the three groups (*p* > 0.05).

**Table 3:** Result of the mCFSS-DS questionnaire ‘Parent’s perception of their child’s fear’ (highest score in bold and underlined).

How Do You Think Your Child Feels at the Thought of …	GP		SPP		UHB	
	Mean	SD	Mean	SD	Mean	SD
1. Dentists	1.5	0.83	1.8	0.70	2.0	1.30
2. Doctors	1.5	0.69	1.6	0.60	1.8	0.91
3. Having somebody examine his/hers mouth	1.3	0.57	1.8	0.85	1.6	0.76
4. Having to open the mouth	1.1	0.22	1.4	0.68	1.3	0.72
5. Being touched by dentist or nurse	1.3	0.44	1.5	0.83	1.4	0.75
6. Having somebody look at him/her	1.3	0.47	1.5	0.51	1.5	0.76
7. The dentist checking his/hers mouth	1.5	0.89	1.8	0.72	1.6	0.82
8. The sight of the dentist checking somebody’s mouth	1.2	0.41	1.3	0.57	1.2	0.52
9. Noise during treatment	1.8	0.64	2.1	0.85	2.0	0.76
10. Having somebody put instruments in his/hers mouth	1.8	0.83	2.1	0.79	2.1	0.94
11. Getting water in his/hers mouth during treatment	1.2	0.52	1.6	0.68	1.4	0.60
12. Having to go to the hospital	**3.1**	1.15	**2.5**	1.54	**2.6**	1.31
13. People in white uniform	1.3	0.44	1.4	0.49	1.3	0.44
14. Having dentist or nurse cleaning his/hers teeth	1.4	0.50	1.8	0.77	1.2	0.37

GP: general practice; SPP: specialized pediatric practice; UHB: university hospital Bonn. There were no significant differences between the three groups (*p* > 0.05).

**Table 4:** Comparison of children’s and parents mCFSS-DS scores with respect to a mCFSS-DS of 30 as threshold for low or high anxiety.

		mCFSS-DS_c_ * n = 60		mCFSS-DS_p_ *^,#^ *n* = 60		Parents’ Own Fear ^#^
	*n*	Mean (SD)	95% CI	Mean (SD)	95% CI	Mean (SD)
LA_c_ vs. LA_p_	36	22.7 (4.61)	21.16–24.28	19.6 (4.28)	18.19–21.09	1.7 (1.14)
LA_c_ vs. HA_p_	3	20.3 (4.93)	8.08–32.59	32.3 (2.31)	26.60–38.07	1 (0)
HA_c_ vs. LA_p_	13	34.9 (6.36)	31.08–38.76	20.8 (3.58)	18.68–23.01	1.5 (0.66)
HA_c_ vs. HA_p_	8	34.3 (4.40)	30.57–37.93	34.6 (4.98)	30.46–38.79	1.8 (1.04)

LA: low anxiety (mCFSS-DS < 30), HA: high anxiety (mCFSS-DS ≥ 30). Subscript: c = child, p = parent. 95% CI: 95% confidence interval. * Significant correlation between children’s mCFSS-DS and parent’s mCFSS-DS (*p* < 0.01). ^#^ Significant correlation between parents’ mCFSS-DS and parent’s own fear (*p* < 0.01). There was no significant correlation between parents’ own fear and children’s mCFSS-DS (*p* > 0.05).

**Table 5:** Children’s anxiety and parents’ perception of their child’s anxiety by a threshold of mCFSS DS > 30 (high anxiety).

	*n* Children/Parent Pair (*n*)	Prevalence (%)	PPV (%)	NPV (%)	Agreement Children’s vs. Parent’s Results (*n*)
GP	20	25	11.8	0	18/20
SPP	20	40	26.7	20	15/20
UHB	20	40	41.2	66.7	11/20
All	60	35	26.5	27.3	44/60

GP: general practice; SPP: specialized pediatric practice; UHB: dental clinic of University Hospital, Bonn, Germany. PPV: positive predictive value; NPV: negative predictive value.

**Table 6:** mDFSS-DS scores in relation to age and gender.

	All			Boys			Girls		
	*n*	Mean	SD	*n*	Mean	SD	*n*	Mean	SD
6 years	9	33.7	10.6	3	39.0	2.6	6	31.1	12.4
7 years	13	22.4	6.8	7	20.7	7.2	6	24.6	6.1
8 years	8	30.5	5.8	5	31.6	6.1	3	28.6	6.1
9 years	8	26.6	4.6	3	24.3	6.5	5	28.1	3.1
10 years	13	23.8	5.6	8	24.2	6.6	5	23.2	4.1
11 years	2	22.5	2.1	1	21.0	-	1	24.2	-
12 years	6	27.0	6.3	3	30.3	6.1	3	23.6	5.5
13 years	1	36.0	0	1	36.0	0	0	0	0
14 years	0	0	0	0	0	0	0	0	0
total	60	26.7	7.6	31	26.9	8.1	29	26.6	7.2

There were no significant differences between mCFSS-DS and age (*p* > 0.05) or gender (*p* > 0.05)

**Table 7:** Distribution of dmft/DMFT in the 3 centers.

	GP		SPP		UHB		Total	
	Mean	SD	Mean	SD	Mean	SD	Mean	SD
dmft *	0.8	1.36	1.0	1.65	4.3	3.93	2.0	2.99
DMFT	0.3	0.91	0.8	0.91	0.75	1.51	0.63	1.31

GP: general practice; SPP: specialized pediatric practice; UHB: university hospital Bonn. * There was a significant difference between the three centers for dmft (*p* = 0.001), but not for DMFT (*p* > 0.05).

**Table 8:** Distribution dmft/DMFT as to age.

	6–7 Years		8–9 Years		10–11 Years		12–14 Years	
	Mean	SD	Mean	SD	Mean	SD	Mean	SD
dmft	3.3	4.0	1.3	2.12	1.7	2.09	0.3	0.49
DMFT	0.4	1.0	0.6	0.96	0.8	1.42	1.3	2.36

There were no significant differences between age and dmft (*p* > 0.05) or DMFT (*p* > 0.05).

## Data Availability

The data that support the findings of this study are available from the corresponding author upon reasonable request.
